# Udder Ultrasonography of Dairy Cows: Investigating the Relationship between Echotexture, Blood Flow, Somatic Cell Count and Milk Yield during Dry Period and Lactation

**DOI:** 10.3390/ani13111779

**Published:** 2023-05-26

**Authors:** Konstantinos S. Themistokleous, Iraklis Papadopoulos, Nikolaos Panousis, Antonios Zdragas, Georgios Arsenos, Evangelos Kiossis

**Affiliations:** 1 Clinic of Farm Animals, Faculty of Veterinary Medicine, Aristotle University of Thessaloniki, 546 27 Thessaloniki, Greece; panousis@vet.auth.gr (N.P.); ekiossis@vet.auth.gr (E.K.); 2 Biostatistics Unit, Quartier Hospital, University of Liège, CHU B23, 4000 Liège, Belgium; ipapadopoulos@uliege.be; 3 Veterinary Research Institute, National Agricultural Research Foundation of Thessaloniki, 570 01 Thermi, Greece; zdragas@vri.gr; 4 Laboratory of Animal Husbandry, Faculty of Veterinary Medicine, Aristotle University of Thessaloniki, 541 24 Thessaloniki, Greece; arsenosg@vet.auth.gr

**Keywords:** milk vein, spectral Doppler, udder echotexture, somatic cell count, milk production, ultrasound, Holstein dairy cows

## Abstract

**Simple Summary:**

Udder health is important for dairy cows’ productivity and welfare. Ultrasonography has been proven a practical method to examine udder health on farms. In this study, we investigated the relationship between ultrasonographic features and indicators of udder health and productivity. Twenty-one Holstein cows were examined repeatedly during a crucial period starting from the end of the late lactation stage, continuing throughout the dry period and ending in the early stage of the consecutive lactation period. Statistical models presented significant associations between blood flow in the milk vein and udder echotexture parameters with daily milk production and milk somatic cell count. Udder ultrasonography is a useful and practical tool for the comprehensive assessment of udder health.

**Abstract:**

Udder health of dairy cows is related to their productivity and welfare. The period from dry-off to calving and early lactation is crucial. Ultrasonography is a useful and practical tool for the examination of the mammary parenchyma and blood flow. This observational study investigated the relationship between udder echotexture features, blood flow volume (BFVol) in the milk vein, milk somatic cell count (SCC) and daily milk yield (DMY) from late lactation, throughout the dry period and consecutive early lactation. Seventeen repeated measurements were performed on twenty-one Holstein cows. The udder parenchyma was examined with B-mode ultrasonography. Udder echotexture was studied using 15 features: Numerical Pixel Value (NPV), Pixel Standard Deviation (PSD), Skewness, Excess, Contrast, Homogeneity, Correlation, Entropy, Run Percentage, Long-Run Emphasis, Grey Value Distribution, Runlength Distribution, Gradient Mean Value, Gradient Variance and Percentage of Non-zero Gradients. Blood flow in the milk vein was examined with spectral Doppler. Linear mixed-effects models were employed to investigate relationships between BFVol, udder echotexture features, SCC and DMY throughout the study period. Our models showed that a 1 kg increase in DMY was associated with a significant increase of 0.25 L/min in the expected BFVol and that a 1,000,000-cells/mL increase in SCC was associated with a significant BFVol decrease of 0.49 L/min, keeping all other variables constant. Multivariable models showed significant associations between DMY and NPV, between PSD and Long-Run Emphasis, and between SCC and NPV, PSD, Gradient Mean Value, Homogeneity, Gradient Variance and Entropy. In conclusion, udder echotexture and BFVol in the milk vein are related to SCC and milk yield. Ultrasonography can be used for the comprehensive assessment of udder health in support of precision dairy farming.

## 1. Introduction

Ultrasonography is an easy, non-invasive method to visualize the udder parenchyma and blood flow on farms [1]. First, amplitude-mode (A-mode) ultrasonography was used to examine internal structures of the udder. Two decades later, brightness-mode (B-mode) was employed to visualize the bovine udder and teat [2]. Ever since, the physiological appearance of the glandular parenchyma and specific ultrasonographic findings under pathological conditions have been reported [3,4,5]. Ultrasound has also been used to assess parenchymal development in dairy heifers [6,7]. Finally, the applicability of three-dimensional ultrasonography to the examination of the bovine udder has been tested and produced good-quality perspective images of the mammary gland [8]. 

Echotexture analysis of udder B-mode sonograms has been used to evaluate mammary parenchyma and increase the acquired information. Echotexture refers to the appearance, structure and arrangement of the parts of an object within an ultrasonographic image [9]. Echotexture analysis employs statistical features, such as mean Numerical Pixel Values (NPV) and Pixel Standard Deviation (PSD)—which are first-order echotexture features—to quantify parenchymal changes depicted in grey-scale images (B-mode sonograms) due to the presence of milk or under pathological conditions [10,11]. In buffaloes, first-order echotexture features were significantly correlated with milk somatic cell count (SCC) [12]. Furthermore, second- and higher-order echotexture features have been used in udder echotexture analysis [13]. These features take into account the position of a pixel, as well as its spatial relationship with its neighboring pixels, and reveal the spatial organization of the image echotexture [14,15,16]. Recently, a deep learning algorithm was trained to process udder echotexture features and predict productivity traits of dairy cows [17].

Blood flow of the udder has been studied with invasive techniques [18,19,20] and with non-invasive color Doppler ultrasonography [21,22,23]. The milk vein (subcutaneous abdominal vein) is the main route of blood drainage from the udder. It plays a key role in the mammary function and is commonly used for drug administration [24,25]. The morphology and blood flow of the milk vein have been studied with color Doppler ultrasonography in lactating Brown Swiss cows [25,26]. Another study compared blood flow of cows in the dry period (DP), cows with 10 kg of daily milk yield (DMY) and cows with 20 kg of DMY and found that milk yield has a profound effect on blood flow variables of the milk vein [27].

Research has shown that udder health and productivity traits affect blood flow and echotexture. Despite being the breed with the highest milk production and worldwide spread, only a few studies have focused on Holstein cows’ udder echotexture, blood flow and morphological parameters of the milk vein [11,28]. There are also limited longitudinal data on changes in udder echotexture features and blood flow of the same cows throughout the period of late lactation, dry-off, udder involution during the DP, calving and early lactation. Moreover, in most studies, only first-order statistics (NPV and PSD) have been used to quantify udder echotexture. According to recent developments [13,17], second- and higher-order statistics could optimize echotexture analysis of the mammary gland, but further research on this topic is needed.

Finally, there is a lack of a comprehensive approach to the ultrasonographic evaluation of udder health and mammary function in the same study. Such an approach could lead to statistical models taking into account the relationships between udder parenchymal echotexture, blood flow, milk SCC—as an indicator of subclinical mastitis—and milk production. This comprehensive approach could be a valuable contribution to clinical practice, as it could aid the data-driven assessment of mammary parenchyma and blood flow, and the detection of alterations due to pathological conditions. Utilizing this approach, veterinarians and farmers could apply precision management strategies to udder health and productivity, based on the ultrasonographic evaluation of the mammary function.

This longitudinal study of clinically healthy Holstein dairy cows, starting from late lactation, continuing throughout dry period and ending in early lactation, aimed to investigate the following: (i) changes in morphological and blood flow parameters of the right and left milk vein, (ii) udder echotexture changes with the employment of first-, second- and higher-order statistics, and (iii) the relationships between blood flow volume in the milk vein, udder echotexture features, milk somatic cell count and milk production.

## 2. Materials and Methods

### 2.1. Animals

The study was approved by the Research and Ethics Committee of the Faculty of Veterinary Medicine, Aristotle University of Thessaloniki (protocol number: 1399/05-04-2019). All operations were conducted in accordance with the EU Directive 2010/63/EU for animal experiments and the university’s guidelines for animal research. Reporting of this observational study was conducted under STROBE-Vet reporting guidelines.

In total, 23 clinically healthy, lactating, pregnant Holstein dairy cows (11 primiparous and 12 multiparous) were initially selected from one commercial herd located in the region of Thessaloniki, Greece. Cows in late and early lactation stages were housed in the same free-stall barn, in the low- and high-lactation groups, respectively. They were milked twice daily with a conventional milking system, and DMY was automatically recorded. They were fed a total mixed ration designed specifically for either the low- or high-lactation group, offered once a day, every morning. Cows in DP were housed as far-off and close-up groups and fed total mixed rations designed for these groups, offered every morning. Detailed information on the nutritional content of the rations is provided in Supplementary Materials.

Median DMY and interquartile range (IQR) at the peak of lactation was 47.3 (10.3) kg, and on the last day of lactation, it was 24.3 (8.5) kg. Median duration of the dry period (IQR) was 56 (6) days. Abrupt dry-off with blanket dry cow treatment was applied using an intramammary antibiotic suspension (Nafpenzal^®^, Intervet International B.V., Boxmeer, The Netherlands) (300 mg procaine benzylpenicillin, 100 mg nafcillin and 100 mg dihydrostreptomycin). On entrance day in the study, median age (IQR) of the cows was 4.2 (1.5) years old; 11 cows were at the end of 1st lactation, 8 were at the end of 2nd lactation, 3 were at the end of 3rd lactation, and 1 was at the end of 4th lactation.

### 2.2. Study Design and Clinical Examination

This observational study consisted of 17 repeated measurements performed on each of the 23 cows throughout 3 consecutive production stages starting from late lactation (7 days before dry-off and at dry-off day), continuing during the DP (on the 3rd, 7th, 21st and 35th day of the dry period, and 7 and 3 days prior to calving) and ending in early lactation (on calving day and on the 3rd, 7th, 14th, 30th, 45th, 60th, 75th, and 90th days of the new lactation period). 

All measurements took place in the middle of the time interval between morning and afternoon milking (10:00–11:00 a.m.). A thorough general physical examination was performed prior to any handling [29]. Udder health was assessed clinically, milk was inspected visually for abnormalities, and California Mastitis Test was performed on farm using a commercially available reagent (BOVIVET CMT Liquid, Jørgen Kruuse A/S, Denmark) [30]. On each measurement day, individual cow DMY was obtained from farm data records and quarter milk SCC (qSCC) and individual cow SCC (iSCC) were recorded on farm with DeLaval Cell Counter (DeLaval International AB, Tumba, Sweden). SCC was log-transformed to somatic cell score (SCS) using the formula SCS = 3 + log_2_(SCC/100.000) [31]. 

Cows with clinical symptoms, moderate or strong California Mastitis Test reaction and/or major pathogens isolated from their milk samples were excluded after the appearance of these conditions. Among the 23 cows initially enrolled in this study, 2 cows were excluded due to the appearance of clinical conditions (clinical mastitis and left displaced abomasum), and all were diagnosed promptly and treated successfully. The final number of cows included in the study was 21.

### 2.3. Ultrasonography of the Milk Vein (B-Mode and Spectral Doppler)

Cows were examined at standing position, non-sedated, restricted in a cattle crush and handled gently to minimize stress. Ultrasonographic examination of all cows was performed consistently by the first author. Morphology and blood flow of the milk veins were examined bilaterally with B-mode and spectral Doppler (triplex) ultrasonography, respectively. Cows had normal resting heart rate during the vascular flow examination [32]. A portable ultrasound scanner (MyLab™ OneVET, Esaote S.p.A., Genoa, Italy) was used, equipped with a broad-bandwidth multi-frequency linear transducer (SV3513, Esaote S.p.A., Genoa, Italy; 2.5–10 MHz). B-mode frequency was 10 MHz, scanning depth was 4–6 cm, gain 58% and time-gain compensation in neutral position. 

**Figure 1:**
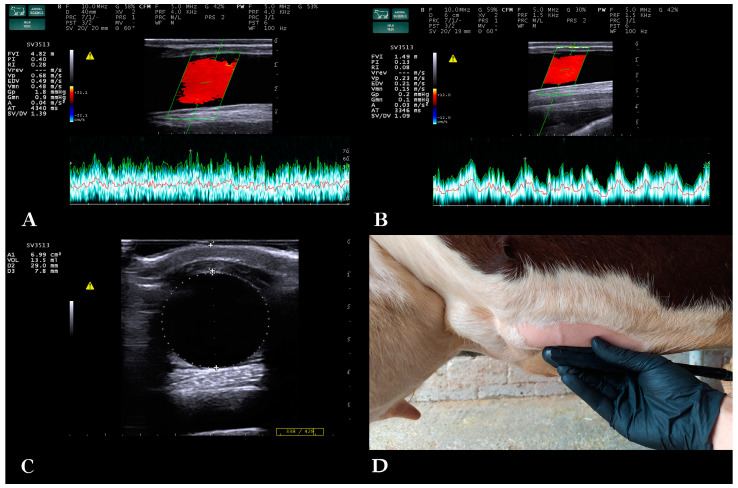
Spectral Doppler examination of the blood flow in the milk vein. (**A**) Measurement of the blood flow features of a dairy cow’s milk vein on the 14th day in lactation, (**B**) measurement of the blood flow features of a dairy cow’s milk vein on the 35th day in the dry period, (**C**) measurement of the distance from skin surface, diameter and cross-sectional area of the milk vein, (**D**) placement of the probe for Doppler measurement of blood flow.

A straight part of the milk vein was selected for measurement located at the midpoint between its cranial and caudal part. The area was shaved, washed, degreased with 70% alcohol and covered with coupling gel (Figure 1). To avoid compression of the vein while ensuring sufficient contact, an appropriate amount of coupling gel was applied on the transducer, without the latter directly touching the skin. First, the transducer was positioned in cross-section of the vein. Distance of the milk vein from skin surface (D1) (cm), its vertical diameter from intima to intima (D2) (cm) and vein area in cross-section (A) (cm^2^) were measured from B-mode images.

Then, the transducer was positioned longitudinally to the vein, and color Doppler gate was activated and placed at the center of the milk vein. Blood flow sample volume cursor included at least 2/3rds of the vein diameter, 16–20 mm wide, and theta angle was 60° (Figure 1). Blood flow was examined for 2 min per vein, and 5 optimal spectral Doppler images were obtained from each milk vein measurement for further processing with MyLab™_Desk v. 9.0 software (Esaote S.p.A., Genoa, Italy). Blood flow parameters used were time-averaged mean velocity (TAMV) (cm/s), peak velocity (Vpeak) (cm/s) and milk vein blood flow volume (BFVol) (L/min) [27]. The total number of spectral Doppler sonograms used was 690 [345 measurement days × 2 milk veins (right/left) per cow].

### 2.4. Ultrasonography and Echotexture Analysis of the Mammary Parenchyma

**Figure 2:**
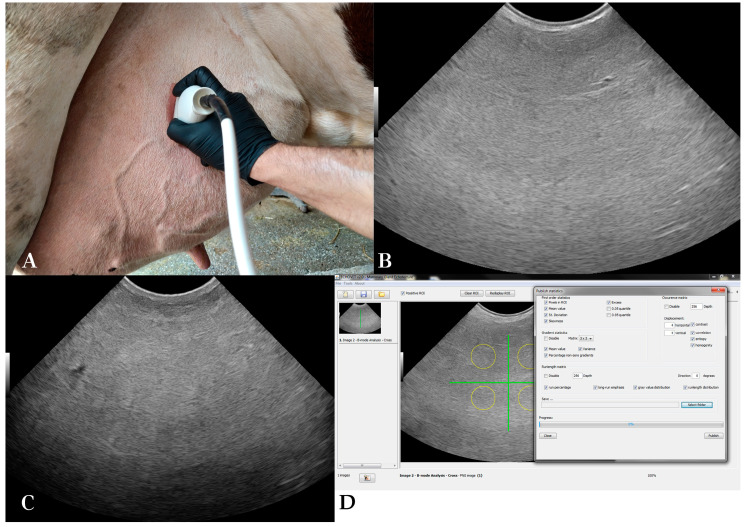
B-mode udder ultrasonography and echotexture analysis: (**A**) Ultrasonographic examination of the mammary parenchyma, (**B**) B-mode sonogram of a cow’s right rear quarter on the 75th day in lactation, (**C**) B-mode sonogram of a cow’s right rear quarter on the 7th day in the dry period, (**D**) echotexture analysis of a B-mode sonogram of the udder (EchoVet v.2.0).

Udder parenchyma was examined with the same ultrasound scanner, equipped with a broad-bandwidth multi-frequency convex probe (SC3421, Esaote S.p.A., Genoa, Italy; 2.5–6.6 MHz). All four udder quarters were examined at each measurement day, and two optimal B-mode images per quarter were acquired. The probe was placed in coronal plane to examine the front quarters and in sagittal plane for the rear quarters, 15–20 cm above the base of each teat (Figure 2). Ultrasound settings remained constant throughout the scans: frequency 6.6 MHz, scanning depth of 15 cm, gain 58% and time-gain compensation in neutral position.

The total number of sonograms used was 2760 (345 measurements × 4 udder quarters × 2 B-mode images obtained from each udder quarter). The ultrasound scanner produced images with a resolution of 720 by 540 pixels. Each pixel had one of the possible shades of grey (0 to 255). Echotexture analysis procedure presented in Figure 2 was performed with EchoVet v.2.0 software (Aristotle University of Thessaloniki, Greece) [13]. Each image was divided into four imaginary quarters [33]. A circular region of approximately 5000 pixels was manually selected within each of the image quarters. Selection criteria were physiological gland parenchyma, with medium homogeneous echogenicity, avoiding mammary vessels [3]. The overall region of interest selected from each B-mode image was 20,000 pixels [17].

Parenchymal echotexture in B-mode images was evaluated using 15 features: mean NPV, PSD, Skewness, Excess, Contrast, Homogeneity, Correlation, Entropy, Run Percentage, Long-Run Emphasis, Grey Value Distribution, Runlength Distribution, Gradient Mean Value, Gradient Variance and Percentage of Non-zero Gradients [17].

### 2.5. Statistical Analysis

Data quality control was performed prior to any analysis. Descriptive analyses were performed to summarize the animals’ characteristics, compared by production stage (late lactation/dry period/early lactation). Quantitative variables were described with means (standard deviation (SD)) and median (IQR). One-way ANOVA for repeated measurements was used to test for differences between production stages. Linear mixed-effects (LME) models under the Maximum Likelihood method [34] were employed to describe the relationship between and the evolution over time of variables of the parenchymal echotexture, BFVol, DMY and SCC, taking into consideration correlations within repeated measurements.

We included random effects (random intercept and slope) for the time of measurement to improve model fitting by accounting for sources of variability that are not explained by the fixed effects in the models. We accounted for individual differences in baseline levels of the outcome variable (individual variability) and for individual differences in the rate of change over time. This is important because individuals may have different trajectories of change over time, even if they started at the same baseline level. Time was included in the models both as fixed and as random effect. Restricted cubic splines with four knots were used to estimate the time evolution curves of udder BFVol, DMY and SCS. The location of the knots was determined based on Harrell’s recommended percentages for a 4-knot RCS [35] with knots at (1) 7 days before dry-off, (2) 36th day of the DP and (3) 30th and (4) 90th day of consecutive lactation. Interactions with time terms were investigated for all models.

All models were minimally adjusted for lactation period and RCS of time to address potential sources of bias due to confounding and capture the outcome’s non-linearity. To improve interpretability of our results, we log_e_ transformed Grey Value Distribution and Runlength Distribution. Additionally, we rescaled Homogeneity, Correlation, Gradient Variance and Long-Run Emphasis values. Model selection procedure with backwards evaluation was performed to determine the final multivariable models, keeping variables with *p* ≤ 0.10. The most significant predictor variables were identified with this method, while controlling for other variables in the models. Model selection was based on the Akaike information criterion and log-likelihood. Multicollinearity was checked for every multivariable model, and collinear variables were excluded based on the highest value of Generalized Variance Inflation Factor [36]. 

The general form of the LME model with a 4-knot RCS that investigated the relationship of BFVol with DMY and iSCC (Supplementary Materials) was as follows:

$$Y_{ij}=RCS{\left(time\right)}_{i}+LP_{i}+iSCC_{ij}+DMY_{ij}+MV\_Side_{ij}+random{\left(intercept,\;time\right)}_{i}+e_{ij}$$

where Y*_ij_* is the BFVol in the milk vein of the *i*th cow at the *j*^th^ measurement day, *RCS*(*time*)_*i*_ is the restricted cubic spline with 4 knots for time for the *i*^th^ cow, *LPi* is the lactation period of the *i*^th^ cow, *iSCC_ij_* is the individual somatic cell count for the *i*^th^ cow at the *j*^th^ measurement day, *DMY_ij_* is the daily milk yield of the *i*^th^ cow at the *j*^th^ measurement day, *MV_Side_ij_* represents the right/left milk vein of the *i*^th^ cow at the *j*^th^ measurement day, *random*(*intercept*, *time*)*_i_* is the random intercept and random slope for time of the *i^t^*^h^ cow and *e_ij_* is the model error for the *i*th cow at the *j*^th^ measurement day.

The multivariable LME model formulas regarding the relationships of DMY with each of the 15 echotexture variables and of qSCS with the 15 echotexture variables are presented in Supplementary Materials. The general form of the LME models with a 4-knot RCS that investigated the relationship of DMY with each of the 15 echotexture variables and the relationship of qSCS with the 15 echotexture variables was as follows:

(1) $$Y_{ij}=RCS{\left(time\right)}_{i}+LP_{i}+Quarter_{ij}+X_{ij}+random{\left[intercept,\;RCS\left(time\right)\right]}_{i}+e_{ij}$$

where Y*_ij_* is the daily milk yield or the quarter somatic cell score of the *i*^th^ cow at the *j*^th^ measurement day, *RCS*(*time*)*_i_* is the restricted cubic spline with 4 knots for time for the *i*^th^ cow, *LPi* is the lactation period of the *i*^th^ cow, *Quarter_ij_* is the udder quarter of the *i*^th^ cow at the *j*^th^ measurement day, *X_ij_* is one of the echotexture variables’ measurement of the *i*^th^ cow at the *j*^th^ measurement day, *random*[*intercept*, *RCS*(*time*)]*_i_* is the random intercept and random slope for time of the *i*^th^ cow and *e_ij_* is the model error for the *i*^th^ cow at the *j*^th^ measurement day.

The analytical versions of the models’ general equations are presented in the Appendix A. Normality of distribution regarding residuals and random effects of the final multivariable models were graphically checked. All *p*-values were two-sided, and significance level was set at *p* ≤ 0.05. All statistical analyses, data manipulation and data visualization were performed using R programming software (version 4.2.2) (R Core Team, 2022). The “lme4” package in R programming language was used to assess convergence of the LME models.

## 3. Results

### 3.1. Descriptive Statistics

**Figure 3:**
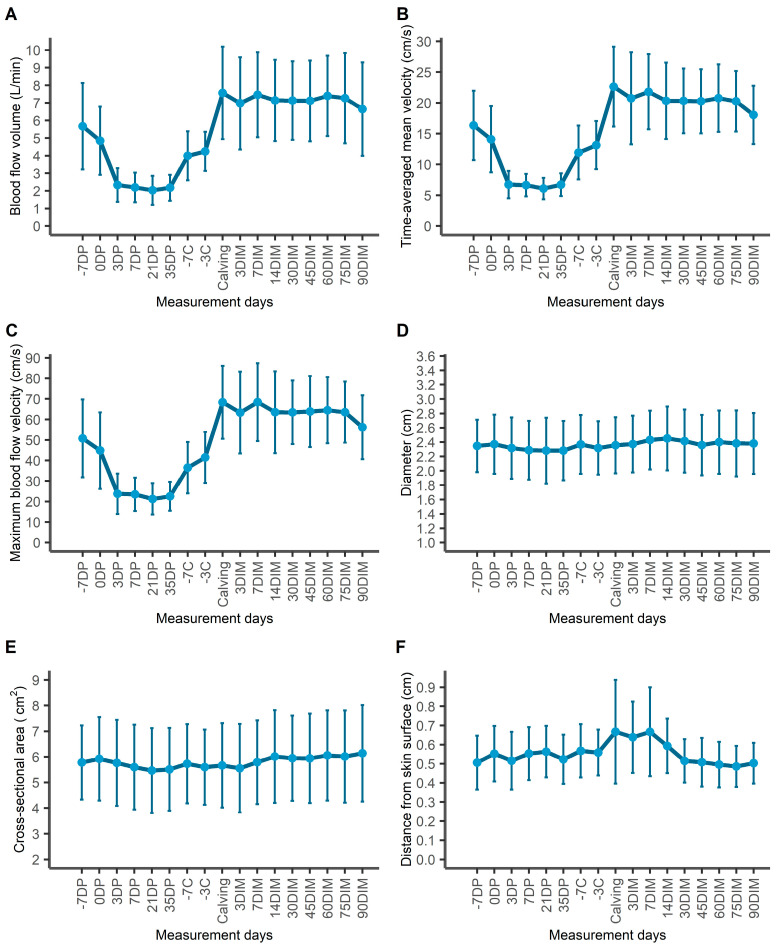
Changes in blood flow (**A**–**C**) and morphological (**D**–**F**) parameters of the milk vein during the study period: starting from 7 days before the dry period (“DP”), throughout the DP, 7 and 3 days prior to calving, at calving (“C”) and from the 3rd to the 90th day in the new lactation period (“DIM”: days in milk).

Repeated measurements from 21 clinically healthy Holstein dairy cows corresponding to 345 cow measurement days were included in our analysis. Changes during the study period in each of the 15 echotexture features used for the investigation of udder parenchymal changes are presented in Supplementary Materials. Morphological and blood flow parameters of the milk vein throughout the study period are presented in Table 1 and Figure 3. Distance of the milk vein from skin surface, its diameter and its cross-sectional area remained constant across production stages.

A significant change was observed in the values of blood flow variables (BFVol, TAMV and Vmax) of the milk vein between late lactation, dry period and early lactation (Table 1, Figure 3). The highest value of mean BFVol was recorded at calving day, at 7.57 ± 2.18 L/min, and the lowest at mid-DP (21st day in the DP). The most abrupt changes in BFVol were observed between the measurements “dry-off day” and “3rd day in DP” (decrease) and between the measurements “3 days before calving” and “calving” (increase). TAMV and Vmax followed the same trend as BFVol throughout the study period. 

### 3.2. Blood Flow Volume, Daily Milk Yield and Somatic Cell Count

The results of the univariable and multivariable LME models investigating the relationship between BFVol in the milk vein, DMY and iSCC are presented in Table 2. A significant association was observed between BFVol and DMY in the respective multivariable model, where side of the milk vein (right/left) appeared significant, too. According to this model, BFVol in the milk vein increases by 0.25 [95% CI (0.13, 0.39)] L/min for every 1 kg increase in DMY, keeping all other variables constant. Lactation period did not significantly affect BFVol in univariable or multivariable models. Individual cow SCC was significantly related with BFVol only in the univariable model. For a 1,000,000-cells/mL increase in iSCC, there is a significant decrease of 0.49 L/min [95% CI (−0.97, −0.01)] in BFVol in the milk vein, keeping all other variables constant. 

### 3.3. Udder Echotexture and Daily Milk Yield

The results of the univariable and multivariable LME models investigating the relationship between udder parenchymal echotexture parameters and DMY are presented in Table 3. Out of 15 echotexture parameters, 6 were significantly related to DMY in the respective univariable models: Mean NPV, PSD, Gradient Mean Value, Correlation, Excess and Skewness. The multivariable model includes 3/15 echotexture parameters: Mean NPV (est. per 1 unit increase (95%CI), 0.09 (0.03, 0.15)), mean PSD (est. per 1 unit increase (95%CI), 0.57 (0.11, 1,03)), and Long-Run Emphasis (est. per 1 unit increase (95%CI), −0.23 (−0.46, −0.002)).

### 3.4. Udder Echotexture and Somatic Cell Score

The results of the univariable and multivariable LME models investigating the relationship between udder parenchymal echotexture parameters and udder quarter SCS are presented in Table 4. 

Out of 15 echotexture parameters, 2 had a significant association with qSCS in the respective univariable models: Mean PSD and Correlation. However, in the multivariable model, 6/15 echotexture parameters were found significant: Mean NPV (est. per 1 unit increase (95%CI), −0.07 (−0.12, −0.02)), PSD (est. per 1 unit increase (95%CI), 0.72 (0.43, 1.00)), Gradient Mean Value (est. per 1 unit increase (95%CI), 0.17 (0.05, 0.30)), Homogeneity (est. per 1 unit increase (95%CI), −1.23 (−2.19, −0.28)), Gradient Variance (est. per 1 unit increase (95%CI), −2.50 (−3.75, −1.26)) and Entropy (est. per 1 unit increase (95%CI), −4.36 (−6.95, −1.79)).

## 4. Discussion

Ultrasonography was employed to examine dairy cows’ udder parenchyma and blood flow repeatedly from late lactation through to the DP and early lactation. This period is very demanding for the udder, as it transitions from a state of milk synthesis to involution and, through intense growth, back to high milk production [37]. This interval is also crucial for udder health because it is related to high infection risk [38]. In this study, we recorded echotextural changes in the mammary parenchyma, as well as changes in morphology and blood flow features of the milk vein throughout the above period. Furthermore, we investigated the associations between udder parenchymal echotexture, blood flow in the milk vein, somatic cell count and milk yield of Holstein dairy cows.

Venous morphological parameters remained constant throughout late lactation, dry period and early lactation stage, despite significant changes in BFVol (Table 1). Change in BFVol is related to milk synthesis taking place in the mammary gland during late and early lactation. Indeed, in our linear mixed model, BFVol appeared significantly associated both with DMY and with production stage (Table 2). This is also apparent in the change in BFVol and DMY over time, as BFVol follows the curve of milk production: a rapid decrease within 3 days after the abrupt dry-off, followed by a stationary (non-lactating) period and then a rapid increase in BFVol 1 week before calving (Figure 3), when parenchymal re-growth and milk synthesis take place. Given the calculation formula BFVol = TAMV × 60 × A [22], the increased blood supply during lactation was achieved by increased flow velocities (TAMV, Vmax), while venous diameter and cross-sectional area remained constant. This explains why BFVol, TAMV and Vmax curves throughout the study period presented almost identical trends.

Our results agree with [22], who observed a significant correlation between DMY and BFVol, and with [27], who found that lactating cows had significantly higher BFVol than dry cows. Brown Swiss cows’ milk vein diameter recorded in [27] was smaller than Holstein cows’ in our study, with no significant difference regarding production status in either study. Even though BFVol of both right and left milk vein did not differ significantly in [27], we included the “side” parameter in our linear mixed model, as it appeared significant. The presence of this parameter in the model formula improves its performance. This is explained by the variability in udder quarter milk yield contribution to the total DMY throughout a lactation period [39], affecting BFVol of each side, too. Finally, mean BFVol values of the milk vein reported by [28] are higher than our findings and those of [26,27]. This could be due to the different technique applied in that study, measuring blood flow in cross-section instead of longitudinal section of the vein. 

A significant association was observed in the BFVol–iSCC univariable model, where measurement day (corresponding to production stage) was significant, too. Cows with high iSCC present lower DMY [40,41,42,43], which in turn is significantly associated with BFVol, as discussed above. The lower DMY and its impact on BFVol could possibly explain the reduced BFVol in cows with high iSCC. Further research could use these clinical observations to distinguish cause and consequence in the BFVol–DMY–SCC relationship and to fully understand how subclinical mastitis affects udder blood flow.

Echotexture of the mammary parenchyma is subject to changes from dry-off, throughout the DP and into regrowth and high lactation [44]. Milk quantity, histomorphology of the udder and ultrasound transducer frequency have an impact on mean NPV and PSD of dairy sheep’s udder sonograms [45]. Milk quantity affects the echotexture features of dairy cows’ udder sonograms, and removal of the hypoechoic milk alters parenchymal echotexture of the mammary gland [11]. In our multivariable model (Table 3), milk yield and production stage were significantly associated with changes in echotexture features (NPV, PSD and Long-Run Emphasis). The variability in milk quantity contained in the mammary parenchyma across different production stages could explain the significant changes in echotexture features throughout the study period. A limitation of our study was the lack of quarter-level DMY data that could aid optimal fitting of the model. Future studies could focus on udder quarter echotextural differences, utilizing quarter milk yield data produced from automatic milking systems to optimize the models’ predictive accuracy.

Normally, udder parenchyma is uniformly echogenic, whereas milk appears anechoic. Mastitis milk has high cellular content, leading to increased milk echogenicity. Subclinical mastitis also leads to increased SCC, and, in chronic cases, it may induce changes in the mammary parenchyma [42], resulting in increased presence of echogenic connective tissue [3,4]. In our multivariable model (Table 4), quarter SCS was significantly associated with NPV, PSD, Gradient Mean Value, Homogeneity, Gradient Variance and Entropy, whilst udder quarter and time were significant, too. The authors of [12] studied buffaloes and also found that echotexture features (NPV and PSD) were significantly affected by SCC, lactation stage, scanning plane and udder quarter. These results indicate that echotexture analysis could aid the detection of subclinical mastitis-induced alterations of the mammary parenchyma. To optimize models’ accuracy, future research should focus on the impact of udder quarter, examination before/after milking, age/lactation period, ultrasound scanning plane and scanner device technical specifications on udder echotexture models.

Our study attempted to connect the dots between udder echotexture and blood flow in the milk vein with milk production and somatic cell count. Based on these results, echotexture analysis could potentially be applied for early diagnosis of subclinical mastitis in lactation, aid the assessment of parenchymal alterations due to chronic subclinical mastitis and suggest a novel method to detect parenchymal alterations due to subclinical mastitis in the DP. Finally, this comprehensive approach to udder ultrasonography, combined with deep learning algorithms [17], could lead to sophisticated models evaluating udder health and productivity in support of precision dairy farming. 

## 5. Conclusions

Ultrasonographic examination of the mammary parenchyma and milk veins is a practical, non-invasive method on farm sites. Blood flow parameters in the milk vein change significantly throughout late lactation, dry period and early lactation, corresponding to lactation needs, while morphological parameters remain constant. To serve these needs, BFVol in the milk vein increases by 0.25 L/min for every 1 kg increase in DMY, keeping all other variables constant. The presence of high SCC in milk is associated with decreased BFVol in the milk vein. Furthermore, second- and higher-order statistical parameters can provide useful information on udder echotexture. Echotexture parameters significantly associated with DMY are NPV, PSD and Long-Run Emphasis. Echotexture parameters significantly associated with SCC are NPV, PSD, Gradient Mean Value, Homogeneity, Gradient Variance and Entropy. These parameters can potentially be used to detect mastitis-related parenchymal alterations and evaluate udder health and productivity, both at individual cow level and at herd level. This comprehensive approach to ultrasonographic examination of dairy cows’ udder echotexture and blood flow can contribute to the data-driven assessment of udder health in support of precision dairy farming.

## Figures and Tables

**Table 1:** Morphology and blood flow of the milk vein stratified by production stage (late lactation, dry period, early lactation).

		Late Lactation (N = 84)	Dry Period (N = 230)	Early Lactation (N = 376)	*p*-Value
D1 (cm)	Mean (SD)	0.53 (0.14)	0.55 (0.14)	0.56 (0.18)	0.287
	Median (IQR)	0.52 (0.23)	0.53 (0.19)	0.52 (0.16)	
D2 (cm)	Mean (SD)	2.36 (0.39)	2.31 (0.42)	2.39 (0.42)	0.591
	Median (IQR)	2.36 (0.52)	2.25 (0.58)	2.37 (0.48)	
A (cm^2^)	Mean (SD)	5.85 (1.53)	5.61 (1.60)	5.90 (1.73)	0.701
	Median (IQR)	5.87 (2.27)	5.44 (2.19)	5.81 (2.00)	
BFVol (L/min)	Mean (SD)	5.26 (2.24)	2.70 (1.30)	7.19 (2.44)	<0.01
	Median (IQR)	4.50 (3.32)	2.40 (1.77)	6.95 (3.42)	
TAMV (cm/s)	Mean (SD)	15.2 (5.60)	8.15 (3.78)	20.6 (5.88)	<0.01
	Median (IQR)	15.0 (10.3)	7.00 (5.00)	20.0 (6.00)	
Vmax (cm/s)	Mean (SD)	47.8 (18.9)	27.10 (11.9)	63.9 (17.6)	<0.01
	Median (IQR)	45.0 (32.2)	24.0 (16)	61.0 (20.2)	

D1: Distance between milk vein and skin surface. D2: Diameter of the milk vein. A: Cross-sectional area of the milk vein. BFVol: Mean blood flow volume of the milk vein. TAMV: Time-averaged mean velocity of the milk vein. Vmax: Mean maximum velocity of the milk vein. N: number of measurements (345 measurement days × 2 milk veins (right/left) per measurement day = 690 in total). SD: Standard deviation. IQR: Interquartile range. *p*-value: One-way ANOVA result between production stages.

**Table 2:** Associations between blood flow volume of the milk vein, daily milk yield and somatic cell count using linear mixed models.

Outcome: BFVol (L/min)	Univariable Model’s Estimates (95% C.I.) ^1^	*p*-Value	Multivariable Model’s Estimates (95% C.I.) ^2^	*p*-Value
DMY (kg)	0.06 *	<0.01	0.25 *	<0.01
	(0.03, 0.08)		(0.13, 0.39)	
iSCC (cells/mL)	−0.49 *	0.04	−0.42	0.07
(for 1,000,000-unit increase)	(−0.97, −0.01)		(−0.91, 0.05)	
Lactation Period (Ref: 1st)				
2nd LP	−0.03	0.948	−0.07	0.913
	(−0.95, 0.90)		(−1.41, 1.25)	
3rd LP	0.41	0.586	0.36	0.725
	(−1.07, 1.87)		(−1.70, 2.41)	
4th LP	−0.75	0.448	−0.41	0.765
	(−2.95, 1.49)		(−4.41, 3.33)	

^1^ All models are adjusted for time; ^2^ Interactions of DMY with time components are included; * statistically significant at the level of *p* ≤ 0.05.

**Table 3:** Association between daily milk yield and udder echotexture parameters using linear mixed models.

Outcome: Daily Milk Yield (kg)	Univariable Model’s Estimates (95% C.I.) ^1^	*p*-Value	Multivariable Model’s Estimates (95% C.I.) ^1^	*p*-Value
Mean Numerical Pixel Value	0.11 (0.05, 0.16) *	<0.01	0.09 (0.03, 0.15) *	0.003
Mean Pixel Standard Deviation	0.72 (0.28, 1.18) *	<0.01	0.57 (0.11, 1,03) *	0.01
Gradient Mean Value	0.30 (0.15, 0.47) *	<0.01		
Contrast	0.007 (−0.01, 0.03)	0.554		
Correlation ^2^	−3.73 (−6.14, −1.48) *	<0.01		
Homogeneity ^3^	−0.86 (−3.13, 1.40)	0.454		
Run Percentage	1.22 (−1.60, 4.04)	0.397		
Gradient Variance ^4^	1.96 (−0.06, 3.98)	0.057		
Grey Value Distribution ^5^	−1.28 (−8.75, 6.18)	0.736		
Runlength Distribution ^5^	−1.16 (−8.72, 6.39)	0.763		
Entropy	0.59 (−4.98, 6.17)	0.835		
Excess	−5.86 (−8.68, −3.05) *	<0.01		
Percentage Non-zero Gradients	−0.46 (−2.29, 1.36)	0.619		
Long-Run Emphasis ^3^	−0.15 (−0.33, 0.02)	0.087	−0.23 (−0.46, −0.002) *	0.048
Skewness	−4.25 (−6.27, −2.23) *	<0.01		

^1^ All models were adjusted for time, udder quarter and lactation period; ^2^ Rescaled × 10^5^; ^3^ Rescaled × 10^2^; ^4^ Rescaled × 10^−3^; ^5^ Log_e_-transformed variables; * Statistically significant (*p* ≤ 0.05).

**Table 4:** Association between udder quarter somatic cell score and udder echotexture parameters using linear mixed models.

Outcome: Quarter Somatic Cell Score	Univariable Model’s Estimates (95% C.I.) ^1^	*p*-Value	Multivariable Model’s Estimates (95% C.I.) ^1^	*p*-Value
Mean Numerical Pixel Value	0.005 (−0.01, 0.02)	0.623	−0.07 (−0.12, −0.02) *	<0.01
Mean Pixel Standard Deviation	0.27 (0.10, 0.44) *	<0.01	0.72 (0.43, 1.00) *	<0.01
Gradient Mean Value	0.04 (−0.02, 0.10)	0.196	0.17 (0.05, 0.30) *	<0.01
Contrast	0.001 (−0.007, 0.010)	0.725		
Correlation ^2^	−1.18 (−2.02, −0.33) *	<0.01		
Homogeneity ^3^	−0.13 (−0.97, 0.71)	0.761	−1.23 (−2.19, −0.28) *	0.01
Run Percentage	0.27 (−0.79, 1.34)	0.615		
Gradient Variance ^4^	0.05 (−0.71, 0.82)	0.890	−2.50 (−3.75, −1.26) *	<0.01
Grey Value Distribution ^5^	0.36 (−2.42, 3.15)	0.798		
Runlength Distribution ^5^	0.49 (−2.32, 3.31)	0.731		
Entropy	−0.72 (−2.82, 1.36)	0.493	−4.36 (−6.95, −1.79) *	<0.01
Excess	−0.52 (−1.57, 0.51)	0.323		
Percentage Non-zero Gradients	0.0005 (−0.68, 0.68)	0.998		
Long-Run Emphasis ^3^	−0.014 (−0.082, 0.053)	0.677		
Skewness	−0.27 (−0.10, 0.46)	0.465		

^1^ All models were adjusted for time, udder quarter and lactation period; ^2^ Rescaled × 10^5^; ^3^ Rescaled × 10^2^; ^4^ Rescaled × 10^−3^; ^5^ Log_e_-transformed variables; * Statistically significant (*p* ≤ 0.05).

## Data Availability

The datasets generated and/or analyzed during the current study are available from the corresponding author on reasonable request.

## Supplementary Materials

The following supporting information can be downloaded at http://dx.doi.org/10.17632/smgvyzy4cp.1. (1) Video S1: color Doppler ultrasonographic examination of the blood flow in the milk vein of a cow. Figures presenting the progress of the 15 echotexture parameters throughout the study period: (2) Mean NPV; (3) PSD; (4) Skewness; (5) Entropy; (6) Run Percentage; (7) Correlation; (8) Runlength Distribution; (9) Contrast; (10) Percentage Non-Zero Gradients; (11) Excess; (12) Gradient Mean Value; (13) Gradient Variance; (14) Grey Value Distribution; (15) Long-Run Emphasis; (16) Homogeneity; (17) Nutritional content of rations.

## Appendix A

We present the analytical versions of the models’ general equations presented in the main text:

1. *Blood flow volume, daily milk yield and somatic cell count*

$$BFVol_{ij}=\beta_{0}+\overset{3}{\underset{k=1}{\sum }}\beta_{k}\cdot S_{k}\left(t\right)+b_{0i}+b_{1i}\cdot Time+\beta_{4}\cdot DMY_{ij}+\beta_{5}\cdot iSCC_{ij}+\overset{8}{\underset{l=6}{\sum }}\beta_{l}\cdot LP_{2\left|3\right|4}+\beta_{9}\cdot MV_{Side}+\overset{12}{\underset{m=10}{\sum }}\beta_{m}\cdot S_{k}\left(t\right)\cdot DMY_{ij}+e_{ij}$$

where *BFVol_ij_* is the blood flow volume in the milk vein for cow *i* at measurement day *j*, $b_{0i}$ is the random intercept for each cow, $e_{ij}$ is the random error of the cow *i* at measurement day *j*, $S_{k}\left(t\right)$, *k* = 1, 2, 3 are restricted cubic splines with four- knot terms, $b_{0i}$ is the corresponding random effect of time, *DMY* is the daily milk yield for cow *i* at measurement day *j*, *iSCC* is the individual cow somatic cell count for cow *i* at measurement day *j*, *LP*_2_, *LP*_3_ and *LP*_4_ are the second, third and fourth lactation periods, *MV_Side* is the left/right milk vein of the *i* cow at measurement day *j* and *β*_10_, *β*_11_, *β*_12_ are the interaction effects.

2. *Udder echotexture and daily milk yield*

$$DMY_{ij}=\beta_{0}+\overset{3}{\underset{k=1}{\sum }}\beta_{k}\cdot S_{k}\left(t\right)+b_{0i}+b_{1i}\cdot s_{1}\left(t\right)+b_{2i}\cdot s_{2}\left(t\right)+b_{3i}\cdot s_{3}\left(t\right)+\beta_{4}\cdot NPV_{ij}+\beta_{5}\cdot LRE_{ij}+\beta_{6}\cdot PSD_{ij}+\overset{9}{\underset{l=7}{\sum }}\beta_{l}\cdot LP_{2\left|3\right|4}+\overset{12}{\underset{m=10}{\sum }}\beta_{m}\cdot Quarter_{B\left|C\right|D}+e_{ij}$$

where *DMY_ij_* is the daily milk yield for cow *i* at measurement day *j*, $b_{0i}$ is the random intercept for each cow, $e_{ij}$ is the random error of the cow *i* at measurement dai *j*, $S_{k}\left(t\right)$, *k* = 1, 2, 3 are restricted cubic splines with four-knot terms, $s_{1}\left(t\right)$, $s_{2}\left(t\right)$, $s_{3}\left(t\right)$ are restricted cubic splines with four-knot terms and $b_{1i}$, $b_{2i}$, $b_{3i}$ are the corresponding random effects, *NPV* is the Mean Value for cow *i* at measurement day *j*, *LRE* is the Long-Run Emphasis for cow *i* at measurement day *j*, *PSD* is the Pixel standard deviation for cow *i* at measurement day *j*, *LP*_2_, *LP*_3_ and *LP*_4_ are the second, third and fourth lactation periods and *Quarter_B_*, *Quarter_C_*, *Quarter_D_* are the quarters of udder.

3. *Udder echotexture and somatic cell score*

$$SCS_{ij}=\beta_{0}+\overset{3}{\underset{k=1}{\sum }}\beta_{k}\cdot S_{k}\left(t\right)+b_{0i}+b_{1i}\cdot s_{1}\left(t\right)+b_{2i}\cdot s_{2}\left(t\right)+b_{3i}\cdot s_{3}\left(t\right)+\beta_{4}\cdot NPV_{ij}+\beta_{5}\cdot PSD_{ij}+\beta_{6}\cdot GMV_{ij}+\beta_{7}\cdot HG_{ij}+\beta_{8}\cdot GradVar_{ij}+\beta_{9}\cdot ENT_{ij}+\overset{12}{\underset{l=10}{\sum }}\beta_{l}\cdot LP_{2\left|3\right|4}+\overset{15}{\underset{m=13}{\sum }}\beta_{m}\cdot Quarter_{B\left|C\right|D}+e_{ij}$$

where *SCS_ij_* is the udder quarter somatic cell score for cow *i* at measurement day *j*, $b_{0i}$ is the random intercept for each cow, $e_{ij}$ is the random error of the cow *i* at measurement dai j, $S_{k}\left(t\right)$, *k* = 1, 2, 3 are restricted cubic splines with four-knot terms, $s_{1}\left(t\right)$, $s_{2}\left(t\right)$, $s_{3}\left(t\right)$ are restricted cubic splines with four-knot terms and $b_{1i}$, $b_{2i}$, $b_{3i}$ are the corresponding random effects, *NPV* is the Mean Value for cow *i* at measurement day *j*, *PSD* is the Pixel Standard Deviation for cow *i* at measurement day *j*, *GMV* is the Gradient mean value for cow *i* at measurement day *j*, *HG* is the Homogeneity for cow *i* at measurement day *j*, *GradVar* is the Gradient Variance for cow *i* at measurement day *j*, *ENT* is the Entropy for cow *i* at measurement day *j*, *LP*_2_, *LP*_3_ and *LP*_4_ are the second, third and fourth lactation periods and *Quarter_B_*, *Quarter_C_*, *Quarter_D_* are the quarters of udder.
